# Painful Muscular Spasm Treated by Excision of Portion of the Nerve-Trunk

**Published:** 1886-02

**Authors:** D. Hayes Agnew


### * Painful Muscular Spasm Treated by Excision of Portion of the Nerve-Trunk.

By D. Hayes Agnew, M. D., LL. D.

*Gentlemen*—Some of you will recognize this patient as the man from whom a portion of the right spinal accessory nerve was excised, towards the close of my lecture one week ago, for a nervous affection, the history of which is briefly as follows: About one year ago he began to experience lateral twitching of the head, with choreic movements of the muscles of the right side of the neck, the sterno-cleido-mastoid and the trapezius being principally involved. This was accompanied by pain, which gradually increased in intensity, so that frequent injections of morphine, hypodermically, had been required for relief. For a short time he was in the medical ward of this hospital, under the treatment of Prof. H. C. Wood. His head was drawn to the right side. Frequent spasmodic rotary motions of the head occurred, and the pain became almost unendurable. Even slight pressure along the right sterno-cleido-mastoid muscle developed

*Clinical Lecture at the University Hospital.

several points of exquisite tenderness and provoked the mo vements of the head. Being guided by the results of pressure, and not by the position of the head, an incision was made along the posterior border of the right sterno-cleido-mastoid muscle, beginning a little below the tip of the mastoid process of the temporal bone, and extending about two and a half inches. After dividing the superficial structures, the posterior border of the muscle was observed; this muscle was then turned towards the median line of the neck, thus exposing the spinal accessory nerve, which principally supplies the sterno-cleido-mastoid and trapezius muscles, and from it about one-half inch of its trunk was excised.

Immediately after the operation all pain and movements ceased, and the patient slept well without an anodyne. Thus far only one twitch of momentary duration has recurred. At present, pressure does not produce pain or motion.

In regard to prognosis, I may say if the irritation has been peripheral the result will be permanent; if central in its origin, the symptoms will return when the nerve extremities unite. Much may also be accomplished by judicious general treatment.—*Medical Times.*

